# Hydrothermal synthesis of nanocrystalline hydroxyapatite–graphene nanosheet on Ti-6Al-7Nb: mechanical and in vitro corrosion performance

**DOI:** 10.1007/s10856-021-06514-w

**Published:** 2021-04-01

**Authors:** Oktay Yigit, Burak Dikici, Niyazi Ozdemir

**Affiliations:** 1 Firat University, Department of Metallurgical and Materials Engineering, Elazig, 23119 Turkey; 2 Ataturk University, Department of Metallurgical and Materials Engineering, Erzurum, 25240 Turkey

## Abstract

The hybrid coatings containing the graphene nano-sheet (GNS) and nano-hydroxyapatite (nHA) phases have been successfully synthesized on Ti6Al7Nb alloys by a one-step hydrothermal method. The hydrothermal reaction was carried out for 24 h at 200 °C. The GNS ratio has been altered as 1, 3, 5 and 7 wt.% in the coatings and, the results have compared with non- GNS containing coatings. The effect of the GNS ratio on the microstructure, hardness, and in vitro corrosion responses has been investigated in detail. The characterizations of the coatings were carried out by SEM, EDS, AFM, XRD and, FTIR. The corrosion behavior of the hybrid coatings was compared in Kokubo’s solution at 37 °C by using potentiodynamic polarization tests. The results showed that the hydroxyapatite phases were deposed on the graphene layers with nano-size nucleation with its Ca/P stoichiometric ratio. The best hydrophilicity (~52°) property has been obtained in nHA/3GNS coatings. In addition, the corrosion rates of coatings increased in the following order: nHA/3GNS < nHA/1GNS < nHA/7GNS < nHA/5GNS < only nHA.

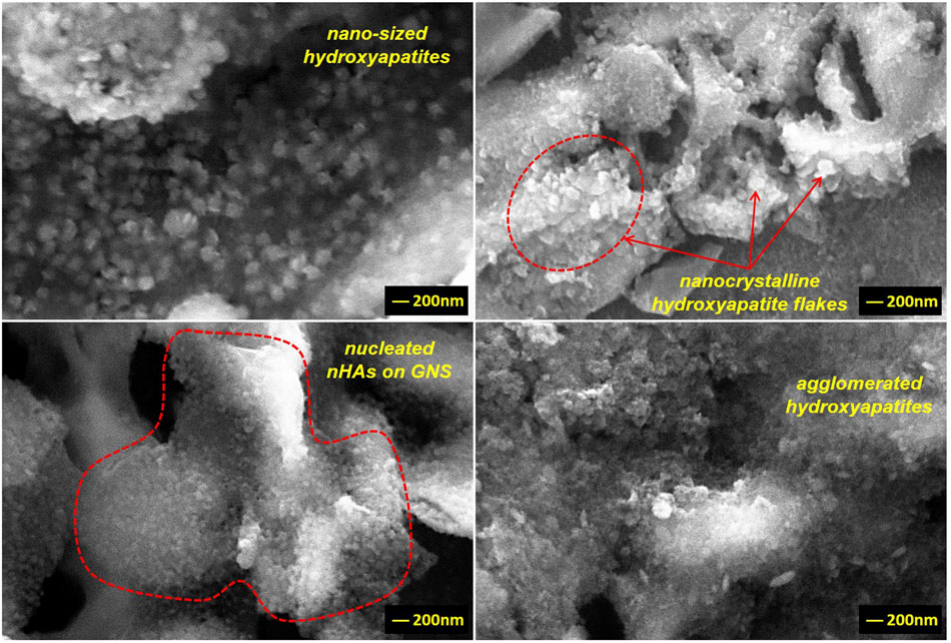

## Introduction

Titanium (CP Ti) and its alloys are preferred in many orthopedic surgeons due to their good bulk properties, high corrosion resistance, and superior biocompatibility although they have low cell adhesion capabilities between the tissue and their surfaces. The most popular Ti alloy using in the biomedical industry is undoubtedly Ti6Al4V [1, 2].

The long-term studies on Ti6Al4V showed that the alloy can cause some health issues. For example, the ion release of vanadium (V) to the body can cause cytotoxic effects and adverse tissue reactions [3, 4]. In recent years, researchers have investigated the progression of new types of Ti alloys such as Ti6Al7Nb or alternative solutions such as bioactive coatings for increasing the osseointegration between the bone/implant interfaces [5, 6]. However, the formation of bone cells on Ti alloys and its ability to bind directly to the bones in the early stages after implantation is very low. Thus, Ca- and P-based coatings are the most common method using increasing the osseointegration on implant surfaces [7].

Superior bioactivity, high cell adhesion and, well cell growth have been observed in Ca- and P-based coatings, especially hydroxyapatite (HA) coatings [7, 8]. The hydroxyapatite (HA, usually written as Ca_10_(PO_4_)_6_(OH)_2_) is a bioactive compound and, like natural bone tissue. Thus, it is often used for bone repair and musculoskeletal tissue regeneration in the field of orthopedic and dental surgery as the coating material. Many studies [9, 10] proved that the HA-coated surfaces have better performance and high biocompatibility than uncoated Ti alloy surfaces. Nevertheless, the low wear resistance and brittleness of HA don’t show the mechanical behavior of the bone structure [11].

Different reinforcements such as TiO_2_ [12], Y_2_O_3_ [13], Al_2_O_3_ [14], SiO_2_ [15], chitosan [16], Ni_3_Al [14] or, carbon nanotubes (CNT) [16] have been added to the HA structure to increase their low mechanical properties until now. Undoubtedly, all of the above increases the mechanical properties of the coatings. However, the reinforcement phases can be also disrupted the bioactivity of the HA and the tissue next to the coatings [17]. Recently, studies on the HA matrix coatings reinforced with carbon-based nanomaterials have increased with increasing technologic progression on the synthesis of carbon-based structure [9, 18]. The main aim of the studies is to progressive the mechanical strength and biocompatibility of HA structure. Therefore, the CNT can weaken the biological activity of HA or cause detrimental influences on the external environment of tissue [17, 19]. However, the GNS can improve both the mechanical properties of HA and increasing its bioactivity. The GNS causes more toughness increase than CNT in the HA structure [20]. Besides, GNS is synthesized in a relatively pure way and, showed much less cytotoxicity on the tissues [17, 21]. By the way, the HA powders can be added to two different sizes as micro and nano in the coating structures. Nowadays, the nanoscale usage of HA is very popular in research studies [22–25]. Nano-size hydroxyapatite (nHA) exhibits high fracture toughness, improved mechanical properties and has a large surface area. Also, rough results showed that nano-HA has better bioactivity than micro-sized HA [24].

Many different methods have been proposed for the graphene additive to the HA matrix in the literature. These are ultrasonic method [26], biomimetic mineralization process [27], radio frequency chemical vapor deposition [28], electro-spin method [29], electrophoretic deposition [17, 18], in-situ synthesis process [30], cold-vacuum spray [31] and hydrothermal method [11, 32]. Nevertheless, any effective way of predicting HA growth on titanium alloys is not available. The hydrothermal method provides many advantages over other methods. It is a method that is less harmful to the environment, has high stoichiometric control, and can be controlled in size, morphology, and agglomeration thanks to its ability to precipitate directly from the suspension. In this way, it is a very successful method in the production of powders in various morphologies and desired sizes. The hydrothermal process appears to be an effective method to achieve HA growth and change the surface structure [23, 33].

Previous studies have generally focused on the effect of graphene additive on the mechanical properties of HA coatings [17, 21, 32, 34]. Besides, the GNS was used in a narrow weight percentage range around 1 wt.% or studied only one additional rate in the works [20, 21, 34].

In this study, the GNSs have been added with wider weight ratios to the HA structure than from the literature by using the hydrothermal method. The nano-sized nucleation of HA particles was provided with the GNS additive. In addition, the effect of the GNS additive on the HA crystallization and the in vitro corrosion properties of the hybrid coatings were tried to understand.

## Experimental procedures

### Material and chemicals

Ti6Al7Nb alloy was used as the substrate for the synthesis of nHA and GNS containing coatings in this study. The chemical composition of the substrate is presented in Table 1. The alloy plates supplied with 50 × 50 × 0.4 cm and then, cut by using a water jet machine with dimensions of 2.5 × 2.5 cm^2^. The cut surfaces were ground with sandpapers step by step from 240 to 1200 to remove the cutting traces formed on their surfaces. Then, the surfaces were ultrasonically cleaned with acetone, ethanol and deionized water, respectively.

**Table 1:** Chemical composition of Ti6Al7Nb alloy (in wt.%)

Al	Nb	Fe	O	N	Ti
6.12	7.07	0.12	0.18	0.01	bal.

Disodium edetate (C_10_H_14_N_2_Na_2_O_8_·2H_2_O), sulfuric acid (H_2_SO_4_, 98%), hydrogen peroxide (H_2_O_2_, 30% aq.), calcium nitrate tetrahydrate (Ca(NO_3_)_2_·4H_2_O), di-ammonium hydrogen phosphate ((NH_4_)_2_HPO_4_) and ammonium hydroxide (NH_4_OH 28% aq.) were purchased commercially from Sigma-Aldrich. Graphene nanosheets (GNS) were purchased from Nanografi Company with 99% pureness. All chemicals were used analytically and without further purification.

### Hydrothermal processes of hybrid coatings

The nHA and GNS containing hybrid coatings were synthesized on the substrates by using a hydrothermal process. Firstly, the substrates were ultrasonically etched for 60 min at 60 °C in piranha solution (H_2_SO_4_: H_2_O_2_ = 7:3, vol/vol ratio). Then, the substrates were fixed vertically in the middle of a Teflon-lined reaction vessel for hydrothermal synthesis. Secondly, Ca(NO_3_)_2_·4H_2_O (0.2 M) with chelating reagent C_10_H_14_N_2_Na_2_O_8_·2H_2_O (0.2 M) solution were prepared in 25 mL deionized water, separately while (NH_4_)_2_HPO_4_ (0.12 M) solution was prepared in 25 mL deionized water. By the way, five different additives of (0, 1, 3, 5, and 7wt.%) GNS was distributed in 10 mL deionized water using an ultrasonic homogenizer (Sonopuls, Bandelin) for 1 h and obtained dark brown well-dispersed solution. After that, all solutions were mixed and the pH of the final solution was raised to 10.5 then mixed with a magnetic stirrer for 60 min at 1000 rpm (Fytronix, FYMS700) to obtain a uniform dispersion. Final suspensions (60 ml) were transferred in a Teflon-lined autoclave (PTFE, 100 ml) for the hydrothermal proses. The hydrothermal processes were performed at 200 °C for 24 h with a hydrothermal reaction system (Fytronix, FYHT-8000). Coated samples were dried in a vacuum oven at 80 °C under 50 mbar pressure for 24 h (Fytronix, HT600). The one-step route for the coatings of nHA/GNS hybrid composites on Ti6Al7Nb has been defined in detail previously in ref. [23].

### Contact (wetting) angle measurements

The wettability properties of the coated surfaces were estimated by contact angle measurements. The contact angle was obtained by using the sessile drop method (Attension® Theta Flex). The simulated body fluid (SBF) tests were used as a solution during the tests. The coated surfaces were also heated at 37 °C for providing in vitro conditions. The wettability angles of the drops on the coating surfaces were measured from the cross-section images recorded by a digital camera.

### Hardness measurements

Surface hardness was measured using by micro Vickers hardness (SHIMADZU HMV-G) technique according to ASTM E384–17 standards [35]. The tests carried out under a 200 gf with a loading time of 15 s. The average hardness was calculated from randomly selected 5 points on each layer, arithmetically.

### In vitro corrosion tests

The electrochemical behaviors of the samples were determined by using the potentiodynamic scanning (PDS) technique under in vitro conditions at body temperature (37 ± 0.5 °C). The potential (*E*) vs. current (*I*) changes of the samples were recorded via a potentiostat unit (Gamry, PCI14/750) in simulated body fluid (SBF). An Ag/AgCl and Pt wire were used as reference and counter electrodes during the tests, respectively. Kokubo’s solution described in ref. [36] was used as the electrolyte. The scan rate of the PDS was 1 mV∙s^−1^. The sample area was about 8 × 8 mm^2^, for this, all data have been normalized to the surface area.

### Characterization

The characterizations of the hybrid coatings were carried out by scanning electron microscope attached to energy dispersive spectroscopy (SEM-EDS, Jeol, JSM-7001F), atomic force microscope (AFM, Park System 100-E), X-ray diffraction (XRD, Bruker D8) and, Fourier-transform infrared spectroscopy (FTIR, Thermo Scientific™ Nicolet™ iS™5) spectroscopy.

## Results and discussion

**Fig. 1:**
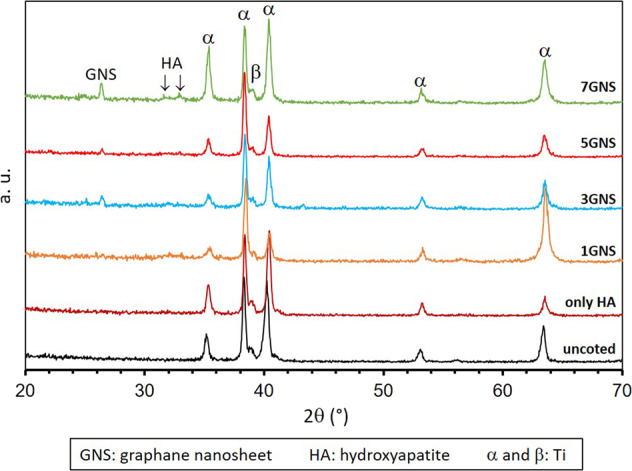
X-ray diffraction (XRD) analysis of uncoated and nHA/GNS hybrid coatings

XRD analysis of nHA/GNS hybrid structures synthesized on Ti6Al7Nb substrates are compared in Fig. 1.

The main hydroxyapatite peaks in composite coatings were observed at 25.8, 32.96° and 35.6° 2theta values with (002), (300), (300) miller indicates, respectively. These refraction peaks are compatible with the standard HA (JCPDS card no. 09–0432) model, suggesting that the synthesized nHA has a high purity crystal structure. The results are in good agreement with refs. [33, 37].

The researchers reported only a weak and wide diffraction peak for GNS at 26.48° of 2theta in nHA/GNS hybrid coatings. However, this large peak is not very obvious because the amount of GNS is not too much and the GNS is spread homogeneously into the coating [38, 39]. The refractive peak of GNS has corresponded to 26.3 in the (002) plane in the present study and, its peak intensity increased while increasing the GNS ratio from 3 to 7 wt.% [20]. All peaks of the pure HA structure described in the literature cannot be seen in the nHA/GNS hybrid coating. Since the GNS additive changes the formation and growth mechanism of nHA nucleation. The study of Neelgund et al. [20] reveals that HA accumulated on graphene is low crystalline and nano-structure. It can be said that the phase formation and crystal structures of Ti6Al7Nb alloys coated by the hydrothermal process can be successfully determined by XRD.

**Fig. 2:**
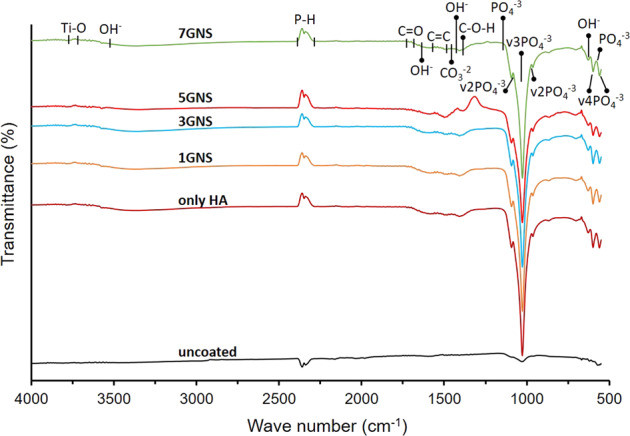
The FR-IR spectrum of uncoated and nHA/GNS hybrid coatings

The FTIR spectra of the nHA/GNS coatings are presented in Fig. 2.

It is well known that the peak positions in the FTIR spectrum related to molecular composition and structure. In FTIR analysis, the band range of 3725–3760 cm^−1^ is matched to the formation of oxygen vacancies induced by the formation of Ti^3+^ ions [32, 40]. Also, the bands in 3540–3730 cm^−1^ attributed to the O–H stretching vibration between hydrogen-bonded molecules of OH glycerophosphate groups of hydroxyapatite [30]. The peak seen between 2340 and 2396 cm^−1^ is associated with the P–H stretching of hydroxyapatite [41]. Wideband between 1678 and 1730 cm^−1^ corresponds to C=O vibration mode in the reports. The O–H bond deformation can be seen at 1630 cm^−1^ [42]. The small broadband in the 1487–1560 cm^−1^ range is assigned to the asymmetric C=C stretching of absorbed water molecules or non-oxidized graphitic fields. In many studies, these very small bands have been reported to confirm the presence of composite coatings and correlate with skeletal vibration mode of GNS due to sp^2^ hybridized C=C vibration stress [17, 20, 21, 43]. The peaks at 1435 and 1407 cm^−1^ are defined as absorption bands. The peak at 1435 cm^−1^ related to O–H deformation vibration and the peak at 1407 cm^−1^ related to C–O–H deformation [32, 43]. During the hydrothermal process, because of the reaction of Ca^2+^ and PO_4_^3−^ ions with each other, the hydroxyapatite phase forms in the coatings. For this reason, PO_4_^3−^ peaks prove the existence of the hydroxyapatite structure. For nHA/GNS composite coatings, a peak at 1028 cm^−1^ and two cantered shoulders peak present at 1095 and 958 cm^−1^ because of the PO_4_^3−^ symmetric stretching mode (v3) and asymmetric stretching mode (v2), respectively. Also, peaks at 601 cm^−1^ and 561 cm^−1^ are characteristic peaks of hydroxyapatite and related to the bending vibration mode of (v4) PO_4_^3−^ [44]. PO_4_^3−^ asymmetric curve bands in 1154, 588, and 561 cm^−1^ can be seen in Fig. 2 [45]. The band at the 630 cm^−1^ is related to the hydroxyl group release mode. The band at the 865 cm^−1^ can be attributed in the reports to the CO_3_^2−^ group vibrations, alongside other characteristic bands of these anions around 1405–1502 cm^−1^. The CO_3_^2−^ bands considered mostly β type hydroxyapatite, which is reported for its superior bioactivity and osteoinductivity, is the prior replacement in bone-like materials [21]. Also, these small amounts of characteristic bands that are containing oxygen between the range of 1300–1600 cm^−1^ are described by a high rate of hydrothermal reactions. According to the previous characteristic peaks of hydroxyapatite and GNS which indicated the nHA/GNS composites have been successfully deposited on the Ti6AL7Nb during the hydrothermal reaction [11, 21, 43]. Also, different GNS additive ratios did not affect the quality of the coating negatively. The FTIR and XRD results appropriate to each other.

**Fig. 3:**
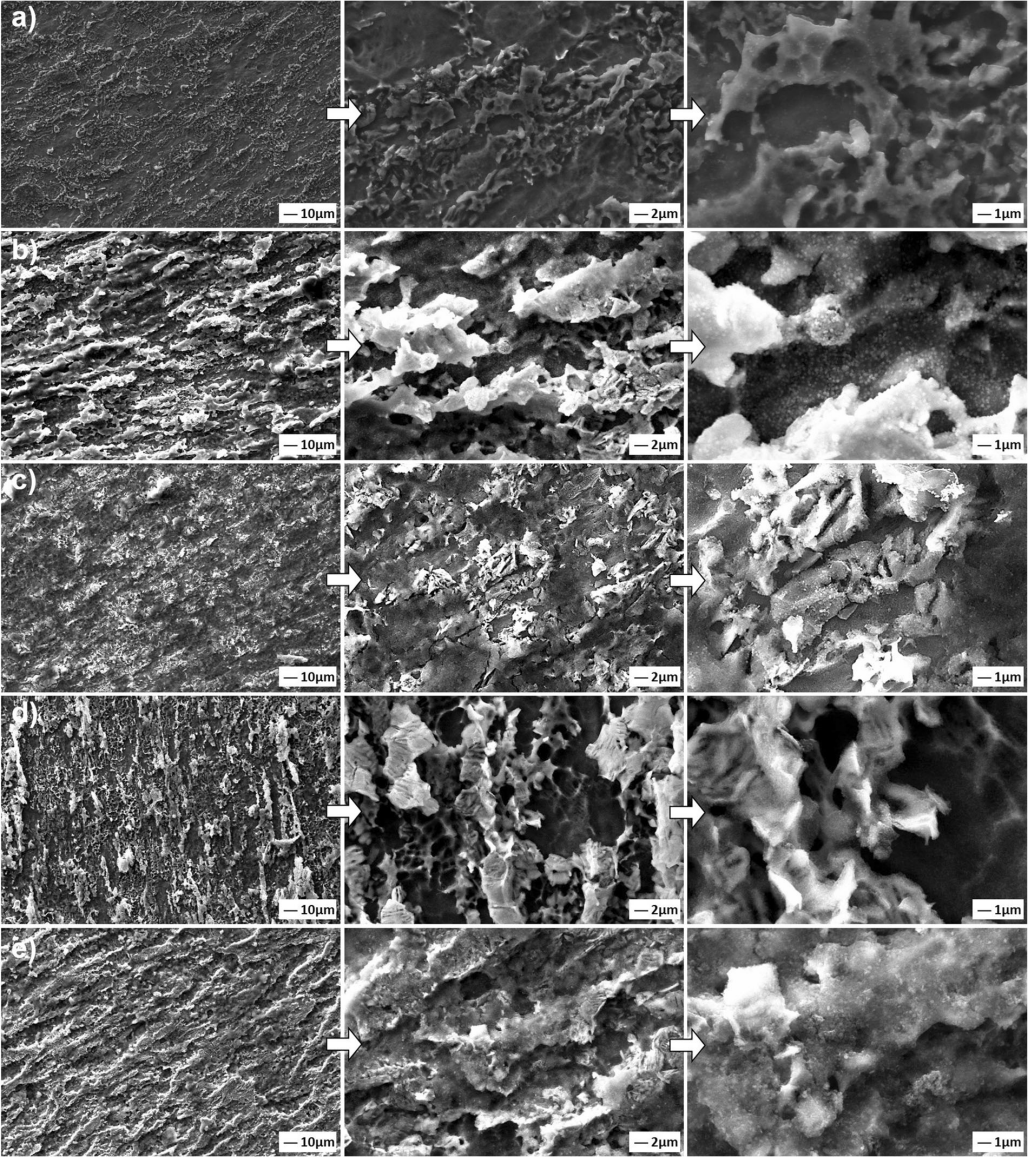
SEM images of coated with (**a**) only nHA, and (**b**) 1, (**c**) 3, (**d**) 5 and, (**e**) 7 wt.% GNS containing structures at different magnifications

The coating morphologies deposited on the Ti6Al7Nb alloys by the hydrothermal method were presented in Fig. 3, comparatively.

A partially porous and non-homogenous morphology was obtained in only HA containing surfaces (Fig. 3a). GNSs were added to obtain a stronger coating layer on the Ti6Al7Nb surfaces and, increasing nucleation points in them. Therefore, the 3 wt.% GNS additive provided the HA particles to expand evenly on the surface of the GNS layers, resulting in slightly cracked brittle coating layers (Fig. 3c). GNS layers are seen in Fig. 3d and e due to their high GNS content. Neelgund et al. [20] reported that GNS interlayers cover a densely packed with nHA nuclei and, forms a sandwich-like layered structure between GR nanosheets and HA. Thus, new and larger nHA agglomerates can be formed in the coating layers as shown in this study (Fig. 3d, e). Similar observations have been also reported by other researchers [26, 46]. The average thicknesses of the coatings were determined as 1.17 (±0.05), 1.70 (±0.12), 2.11 (±0.08), 2.61 (±0.13) and 5.34 (±0.09) µm for the only nHA and, 1, 3, 5 and 7 wt.% GNS containing structures, respectively. Consequently, it can be said that denser and high thickness coating layers can be formed with increasing GNS wt.% in the layers on the Ti6Al7Nb substrates.

**Fig. 4:**
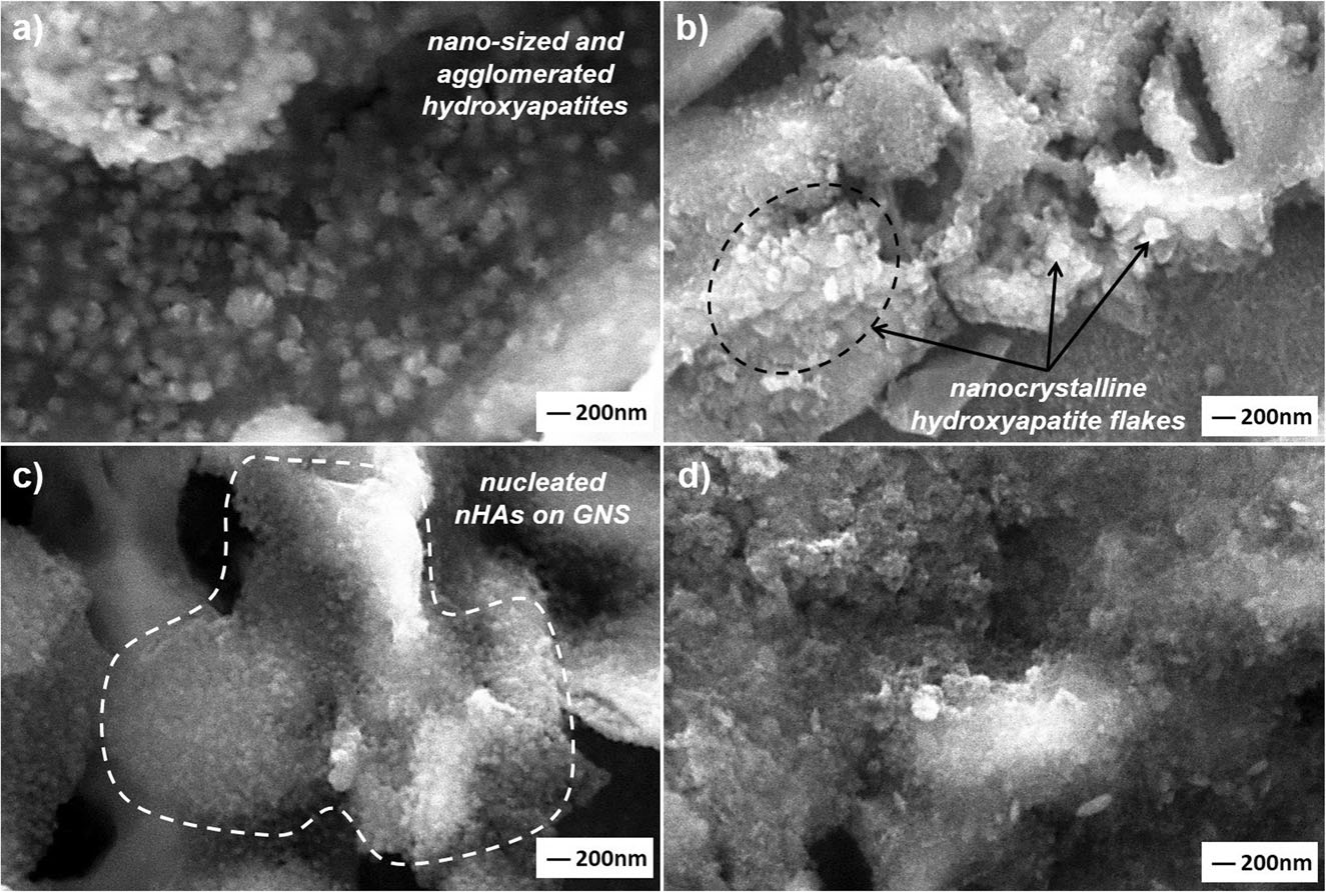
SEM images of the nHA/GNS hybrid coatings at higher magnifications of (**a**) 1, (**b**) 3, (**c**) 5 and, (**d**) 7 wt.% GNS containing structures

SEM images reveal that increasing the GNS wt.% in the layers increases the number of nHA nuclei and surface roughness in the layers and, able to obtained almost an even distribution over the entire surface (Fig. 3). Porosities and protrusions on the coating layers are stood out due to the propagation of nHA particles on GNs layers during the synthesis. The porosities cause a rough on the surfaces. However, the HA structure is not the only factor affecting surface roughness and the amount of porosity in the layer. GNSs, which are vertically and loosely dispersed on the coating surface, contributes to the behavior with nucleated nHA on them. Increasing the GNS ratio in the coating layer forms a more intensive coating layer on the substrates, relatively (Fig. 3e). Thus, the coating surfaces exhibit high porosity and roughness morphology. It was reported that a larger surface area (or roughness) and more porosity are activated osteoblastic cell adhesion to the implant surface and, accelerates its osseointegration [47]. The porosity content has been calculated via the ImageJ analysis program as 11.55, 12.64, 21.04 and 19.90 for the only nHA and 1, 3, 5, 7 wt% GNS containing structures. The highest porosity rate was found in synthesized coating with 5% by weight GNS additive. The porosity content of coating reinforced with 7 wt.% GNS is also close to the sample, but its coating thickness is thinner than the nHA/7GNS wt.% sample. This proves that the coating density increases considerably and, the desired high roughness and porosity ratio on the surface is maintained [25]. Thus, it can be concluded that the porosity increasing is not related to only the GNS additive, it can be also linked with large diameter nHA agglomerates formed on the coating surface. By the way, the arrangement and direction of the GNS on the coating surface is another important factor affecting the surface properties. GNS accumulated in layers provides a more intensive coating due to the nucleation of nHA in the interlayers. The GNSs which are vertically and loosely dispersed on the coating surface ensure are be porous and rough together with the nHA nucleated on them. Thus, denser coatings can be obtained with the addition of GNS while the coating surfaces exhibit high porosity and roughness morphology. However, nHA nucleation has not been observed on the surfaces at these magnifications. Thus, SEM images of the GNS containing layers at higher magnifications have been presented in Fig. 4.

**Fig. 5:**
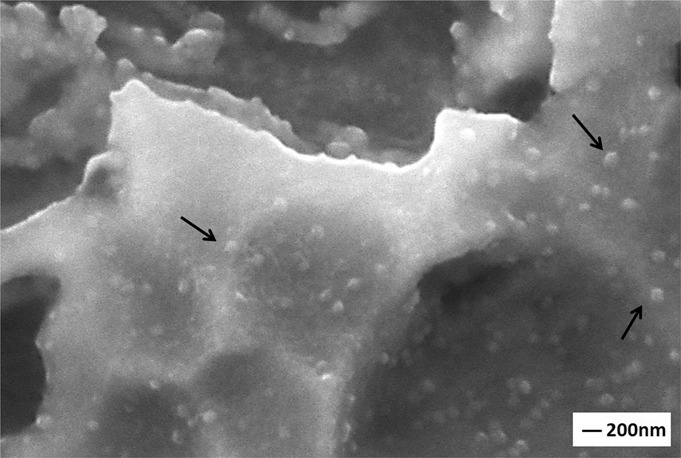
SEM image of only nHA coating at higher magnification

Nucleated HA and their agglomerated structures on the GNS layers (Fig. 4a, c) have been clearly observed on the surfaces and, synthesized nanocrystalline hydroxyapatite has preferentially flake-like structures (Fig. 4b). Therefore, more clear images could not be getting due to the non-conducting structure of nHA although SEM images were taken even at 50.000x magnification. The rod-like structure of nHA was also not observed in this study, probably due to the higher content of GNS in the layers. Of course, the HA grains nucleate on and grow along with GNS as normally. However, Liu et al. [48] reported that GNSs have been inhibited grain growth of HA in at least one direction and, the GNS containing coatings forms finer HA grains. The increasing GNS content inhibits the formation of rod-like nano HA and its grain-size. Thus, the GNSs form serial wall regions insulating particular HA grains so, the nHA grains grow as abnormal. In their study, the maximum GNS ratio was 1 wt.% while the maximum GNS ratio in the layers is 7 wt.% in this study. It can be concluded that the inhibition effect of GNSs is more dominant (Fig. 4d). However, the number (or rate) of nHA nucleation has accelerated significantly with the increment of GNS in the layers compared to only HA coatings (Fig. 5). Some nHA nuclei have been marked with black arrows in Fig. 5.

**Fig. 6:**
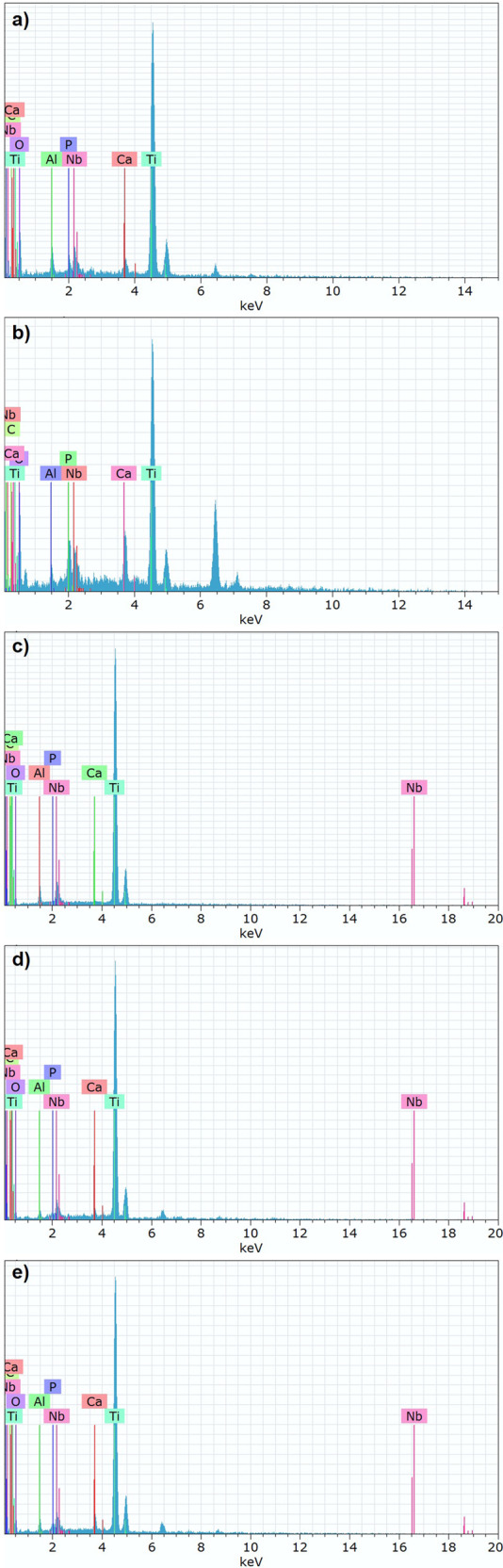
EDS spectrums of the coated samples with (**a**) only nHA, and (**b**) 1, (**c**) 3, (**d**) 5, (**e**) 7 wt.% GNS containing structures

Figure 6 presents the EDS analysis taken from SEM images had 2 µm scale in Fig. 3. The summary of the elemental ratio in the EDS analysis is also collected in Table 2.

**Table 2:** The elemental ratio in the EDS analysis of nHA/GNS hybrid composite on Ti6Al7Nb alloy surface (at. %)

Coating	Ti	Al	Nb	O	Ca	P	C	Ca/P
only nHA	47.08	1.56	1.30	46.65	1.69	1.01	0.71	1.67
nHA/1GNS	30.72	0.74	1.06	58.00	3.67	2.21	3.60	1.66
nHA/3GNS	46.96	1.39	1.36	41.44	1.65	0.96	6.24	1.72
nHA/5GNS	40.43	1.19	1.67	46.57	1.90	1.12	7.12	1.70
nHA/7GNS	40.89	1.06	1.40	45.89	1.79	1.07	7.90	1.67

One of the important advantages of the coatings made by the hydrothermal method compared to other methods is that the Ca/P ratio can be adjusted more easily [32, 49]. The Ca and P compounds added to the solution are adjusted according to the stoichiometric ratio before coating, and there are no major factors that can affect the Ca/P ratio during the coating. Thus, it is easier to catch the 1.67 Ca/P ratios of HA by the hydrothermal method. On the other hand, it is more difficult to control the Ca/P ratio in coatings electrochemically (such as PEO or sol-gel) [50–52]. Since the electron negativities of calcium and phosphate are different. Therefore, the Ca/P ratios on the coating surfaces and in their layers may differ. Also, the Ca/P ratio is highly dependent on the solution properties, the optimization of coating and electrolyte properties is very important in these coatings [53]. The basic components using the preparation of hybrid coatings are confirmed with the analysis. The results indicated that the stoichiometric Ca/P ratio (∼1.67 in HA structure) was almost obtained in all coatings. However, there are some deviations between them due to the representation ratio of coating with the selected area, probably. By the way. the Ca/P ratio of natural bone tissue has been given as 1.67 in most literature [4, 47]. Therefore, the carbon ratio in the coating layers different from the addition ratio due to the polymerization of hydrocarbons during electron bombardment on the surfaces. Control of the coating composition, which is one of the most important advantages of the hydrothermal method, can be determined by EDS analysis. The hydrothermal method has been also concluded to be a very effective method for nHA/GNS hybrid coatings on the Ti6Al7Nb surface [18, 23]. It should be reminded that it is very difficult to obtain a reliable elemental analysis of compounds to an accuracy better than 1% absolute with EDS in many cases.

**Fig. 7:**
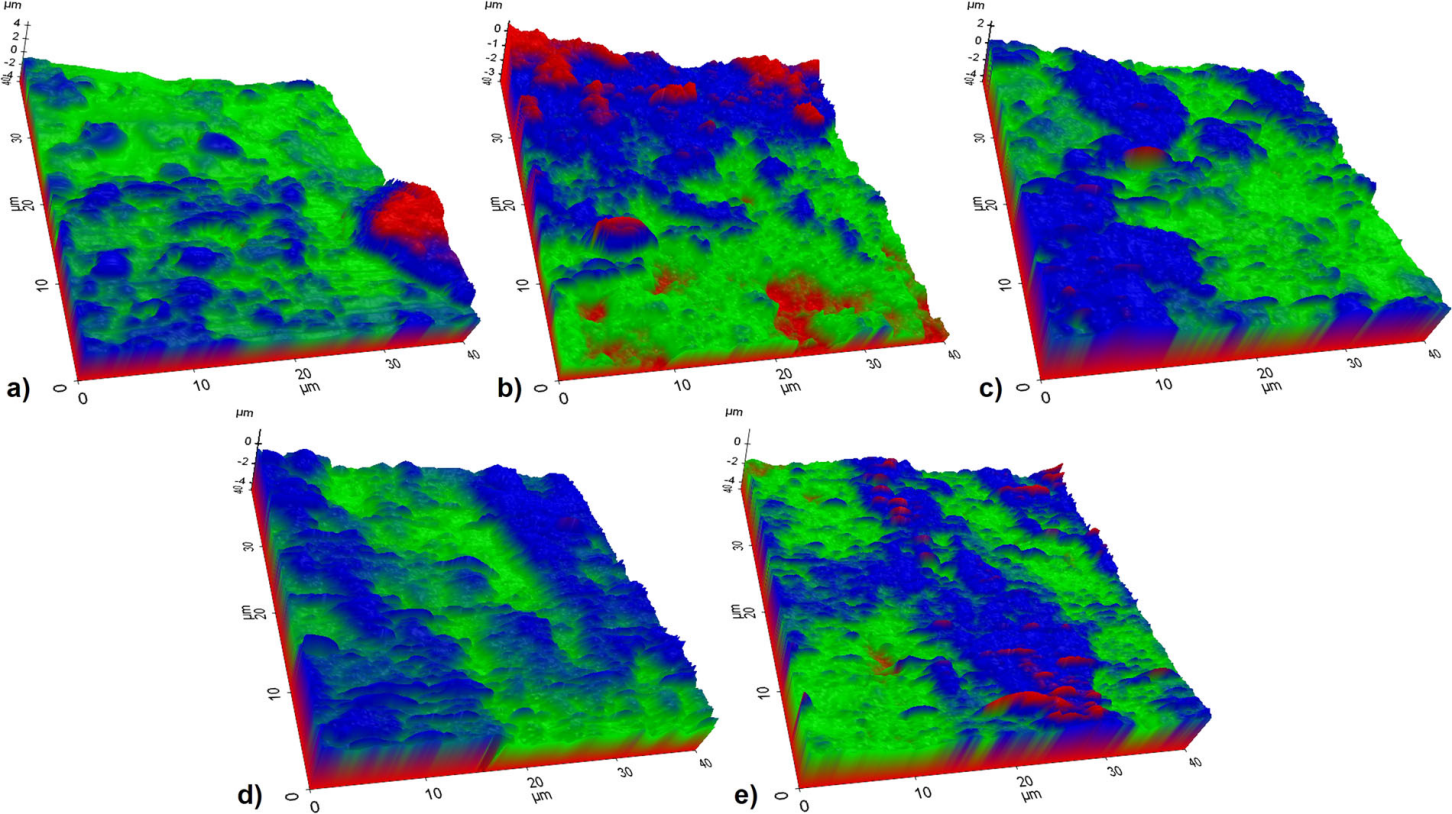
AFM images of the coated samples with (**a**) only nHA, and (**b**) 1, (**c**) 3, (**d**) 5, (**e**) 7 wt.% GNS containing structures

Figure 7 shows 3D topographic surface AFM images of hybrid coatings on Ti6Al7Nb. Besides, the surface roughness parameters of AFM images were collected in Table 3.

**Table 3:** The surface roughness parameters of AFM images

Parameter	only nHA	nHA/1GNS	nHA/3GNS	nHA/5GNS	nHA/7GNS
Min (μm)	−4.758	−3.561	−4.758	−4.758	−4.758
Max (μm)	4.596	0.644	2.463	1.344	1.494
Mid (μm)	−0.081	−1.458	−1.148	−1.707	−1.632
Mean (μm)	−1.004	−1.647	0.001	−0.899	−0.806
R_pv_ (μm)	9.354	4.205	7.222	6.102	6.253
R_q_ (μm)	0.951	0.741	0.682	0.597	0.455
R_a_ (μm)	0.615	0.608	0.570	0.487	0.365
R_z_ (μm)	9.344	4.117	6.927	5.816	4.190
R_sk_	−2.104	−0.123	−0.202	0.199	−0.250
R_ku_	10.463	2.510	2.920	3.204	3.452
S_a_ (µm)	1.133	1.150	0.570	0.921	0.823
S_q_ (µm)	1.283	1.305	0.682	1.078	0.925
Surface area (µm^2^)	2763	2496	2587	2572	2425

It was observed that NHA nanoparticles on GNS layers were distributed almost evenly on the substrate material in all coatings. It is well known that R_a_ and R_q_ give the roughness values in a line. S_a_ has defined the extension of R_a_ (the arithmetic mean height of a line) to a surface and, and S_q_ has defined the extension of R_q_ to the field (the mean square root value of ordinate values in the defined area) and represents. These parameters are frequently used to appraise surface roughness [54].

There is a significant decrease in the roughness of the coatings with the addition of GNS according to Table 4. S_a_ and S_q_ values of the only nHA and nHA/1GNS coatings were especially higher than that of the other coatings. Similarly, R_a_ and R_q_ values are also higher than the others. The lowest surface roughness was obtained in coatings with 3 wt.% GNS additives. However, it is seen that the surface roughness increases again in the samples with 5 and 7 wt.% GNS additives. This high surface roughness may indicate the nonhomogeneous HA structure on the surfaces due to the low GNS additive in the coatings. Thus, a more homogeneous distribution has occurred on the coatings reinforced with 3, 5, and 7 wt.% GNSs. Since the number of nHA crystals increased significantly with the addition of GNS as seen from SEM analysis (Figs. 3 and 4). Accordingly, the addition of GNS affected positively the coating quality and provided the formation of more homogeneous coatings. Since HA nanocrystals achieve a wider surface area with an increasing GNS ratio in the coating layer, where they can hold on to the coating surface evenly. The binding of nHA to the surface enables also the attachment of osteoblast cells [47, 55].

**Table 4:** Corrosion parameters calculated from PDS curves of the synthesized hybrid coatings on Ti6Al7Nb

Coating	*E*_corr_ (mV)	*I*_corr_ (nA·cm^−2^)	*I*_pass_ (nA·cm^−2^)	Corr. Rate (mpy)	Corr. Rate (nm·yr^−1^)	*R*_p_ (ohms·cm^2^)
only nHA	15.58	135	351	0.084	2.123	102,580
nHA/1GNS	98.00	92	171	0.057	1.447	253,681
nHA/3GNS	28.00	61	128	0.038	0.959	323,392
nHA/5GNS	79.89	114	281	0.071	1.793	176,812
nHA/7GNS	45.00	97	157	0.060	1.526	253,585

**Fig. 8:**
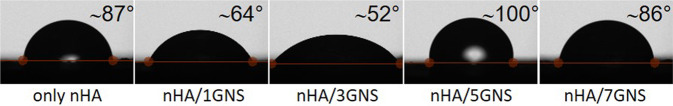
The comparing of the contact angles of the coatings

However, the attachment is also related to the wettability of surfaces. It is well known that there is an inverse relationship between the contact angle and wettability. In other words, a decrease in the contact angle increases the wettability capacity of the surfaces. The measurements of the contact angle of the hybrid coatings are comparatively presented in Fig. 8. All coatings have hydrophilicity character (<90°) except for nHA/5GNS coating as seen from Fig. 8.

The best hydrophilicity (~52°) property has been obtained in nHA/3GNS coatings. Interestingly, the contact angle of the composite coatings increased initially with the increment of the GNS ratio (up to 3 wt.%) and then decreased again. The trending is similar to the surface roughness results described above. It can be easily concluded that there is a relationship between the surface roughness and wettability of the coatings.

**Fig. 9:**
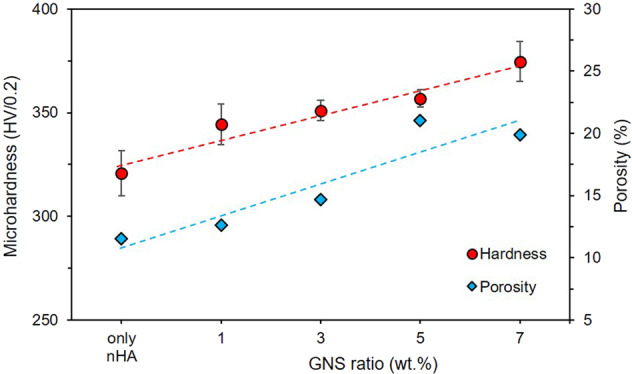
Comparison of the microhardness and porosity values of the coatings, and their trendiness

The mechanical properties of biomaterials in clinical applications is very important for evaluating the working conditions and life of them [56]. Microhardness measurement is a fast and safe way of understanding comparing the layer density and crack mechanisms of the hybrid coatings under load. It is also a widely used technique in surface coatings [38, 57]. A comparison of the hardness and porosity values of the coatings is presented in Fig. 9.

The microhardness value of the coatings increased slightly with increasing of GNS additive to the coating. By the way, there was no significant difference between the uncoated substrate (311.5 ± 10.9) and only the nHA coated sample. The increase in the hardness can be related to increasing the GNS surface area ratio within the coating since nHAs find more settle to form a denser and harder layer in there [20]. Therefore, a less and narrower crack formation under load during the microhardness measurements in GNS doped coatings has been observed than only nHA coatings. It has been reported that nano HA coatings increase the fracture toughness due to the increase in the amount of nano HA in the coating with the GNS additive. It beliefs that a denser coating layer is formed with increasing GNS and, obtained higher fracture toughness in the coating [38, 39]. Besides, the porosities can cause a rough on the surfaces as discussed above sections. Figure 9 confirmed that there is a strong relationship between the hardness and porosity values of the synthesized layers.

**Fig. 10:**
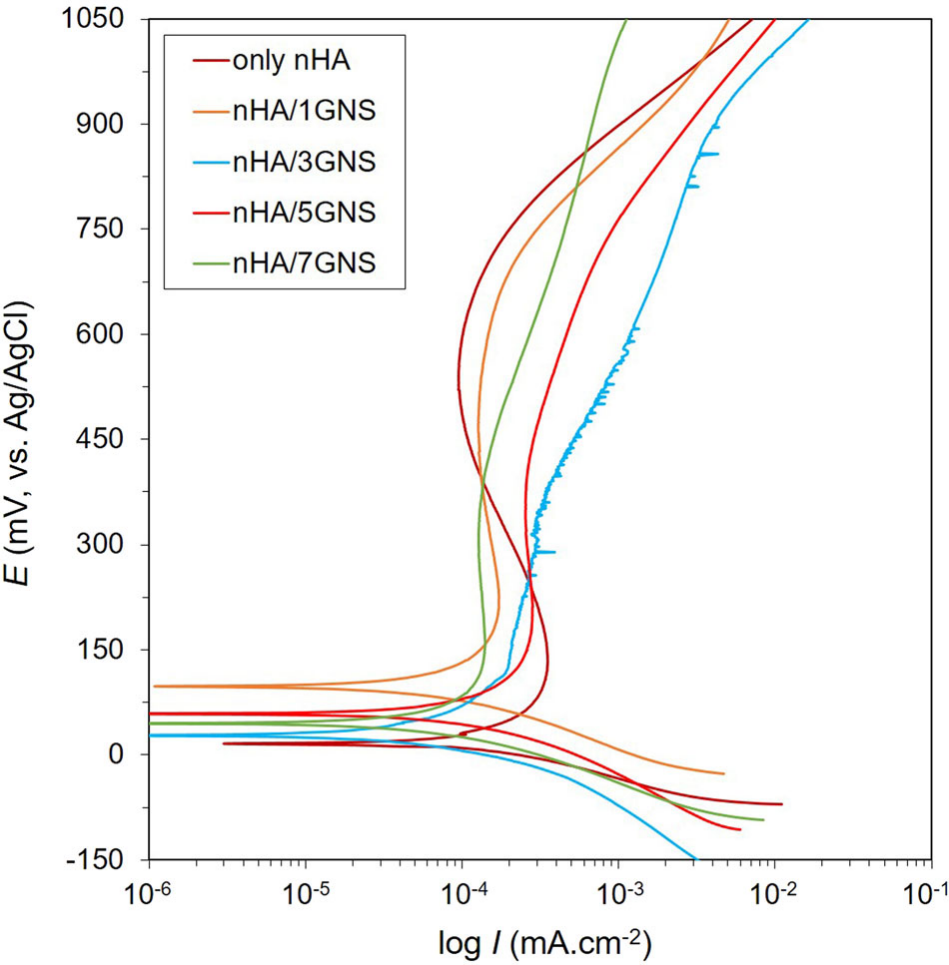
The comparative PDS curves of hybrid coatings on Ti6Al7Nb

The polarization scanning (PDS) of synthesized coatings on Ti6Al7Nb is shown in Fig. 10. Besides, some important corrosion parameters calculated from these curves are given in Table 4.

The lowest and highest corrosion current density (*I*_corr_) values were obtained in nHA/3GNS and only nHA coatings, respectively. Practically, the magnitude of the *I*_corr_ value of the coating indicates its tendency to corrosion. In this study, the corrosion rates of coatings were calculated based on ASTM G102 [58] in both “mpy” and “nm·yr^−1^” ratios as seen in Table 4. The corrosion rates of coatings increased in the following order: nHA/3GNS < nHA/1GNS < nHA/7GNS < nHA/5GNS < only nHA. Although the corrosion rates of all GNS doped coatings were lower than only nHA coating, the *I*_corr_, passivation current density (*I*_pass_) and polarization resistance (*R*_p_) values of 1 and 7 wt.% GNS containing coatings were very close to each other. By the way, the *I*_corr_ values of all coatings during the anodic forward scanning did not change on a large scale when the potential was increased. This is due to the passivation on coated surfaces. The nHA/5GNS structure was presented a higher *I*_pass_ than other GNS containing coatings (Table 4). This indicates that the coating has a higher porosity and heterogeneous structure (Figs. 3 and 9). Since porosities are generally areas where the electrolyte stagnates and these areas are subject to infiltration of Cl^−^, HCO_3_^−^, or HPO_4_^2−^ ions in the SBF, especially. Thus, the *I*_pass_ value of the coating may have been increased.

However, porosity occurring on the surfaces of these coatings causes discontinuities on the protective oxide layer under normal conditions. The porosity content of the coatings increased with the increasing GNS ratio as explained in Fig. 9. The increase in porosity rate is undoubtedly related to the nucleation mechanism, agglomeration, and homogeneity of the HA on GNS layers. Thus, while the GNS ratio of the coating increases, nHA crystals obtain larger surfaces for nucleation. Thus, it can be expected that the corrosion resistance will increase with the increasing GNS rate in the coating layer due to the nucleation of nHA.

**Fig. 11:**
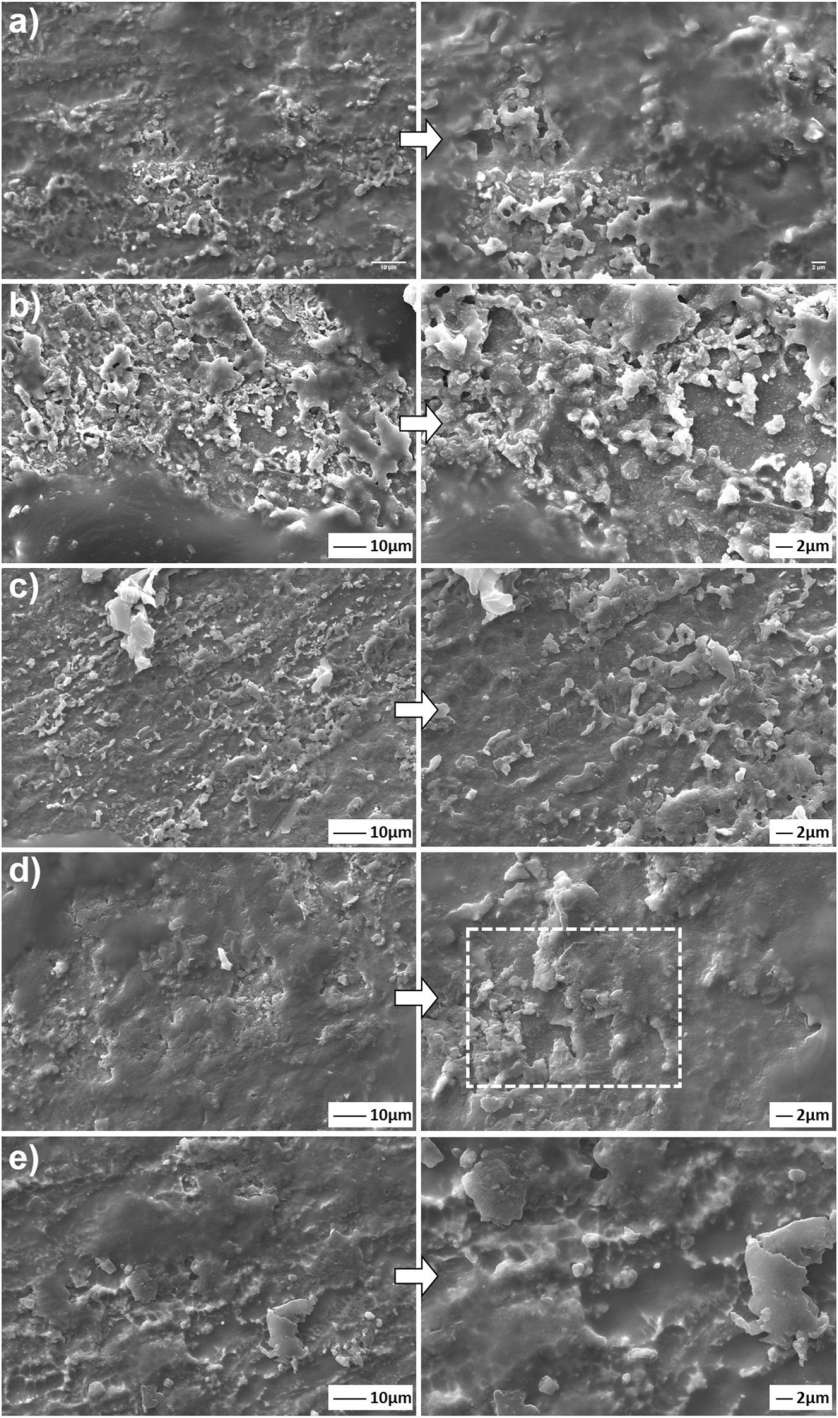
SEM images of corroded surfaces after in vitro corrosion tests in SBF. (**a**) only nHA, and (**b**) 1, (**c**) 3, (**d**) 5, (**e**) 7 wt.% GNS containing structures

**Fig. 12:**
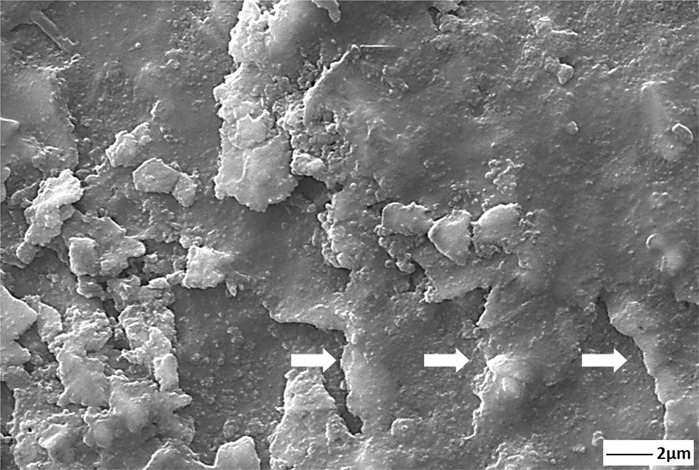
SEM image of the nHA/5GNS hybrid coating at higher magnification the dashed area in Fig. 11d

Therefore, thick and dense GNSs in the coating layer have C=C or C=O groups in the structure, and this structure can preferably limit the electrolyte leaking into the substrate or interfaces between nHA/GNS interfaces [32]. Besides, HA nucleation occurs on the surfaces of the GNS layers as well as on the interfaces. Accordingly, increasing the density of the coatings may prevent the electrolyte from seeping into the interfaces and the substrate surface. The HA has excellent biocompatibility without forming any toxic interaction and, is known as osteoconductive with hard tissues [59]. However, the nHA/5GNS structure in the GNS containing coatings showed a higher corrosion rate than the others. Nevertheless, while the corrosion traces were seen on the only nHA and nHA/1GNS coating structures, the integrity of the coating layer was still protected on the nHA/5GNS sample surface after in vitro corrosion tests (Fig. 11). This contradiction is not an expected situation, normally. Figure 3 showed that the nHA/5GNS structure has larger nHA agglomerates and a wider sandwich-like layered structure between GRSs than others. Figure 9 was also revealed that the structure has a higher porosity content than other coatings. It can be concluded that the porosities are areas where the SBF is stagnating and oxygen transformation is more difficult in there. Thus, the corrosion continues under the layered structures and the around of agglomerated nHAs by increasing the potential due to their nobler structures. Thus, corrosion may have been continued under the layers although the coating layer protects its integrity. The high magnification image (Fig. 12) of the area indicated by the dashed line in Fig. 11d may support the above suggestion. The corrosion paths progressed under the layers were marked by white arrows in Fig. 12. However, it is often not easy to confirm the discussion due to the difficulty of sample preparation from a cross-section of the coatings after corrosion tests.

It can be concluded that there is a critical GNS ratio to provide an in vitro corrosion resistance of the coatings on the Ti6Al7Nb. For the investigated GNS ratios, the 1 or 3 wt.% GNS addition has a predominant role in the corrosion resistance of the coatings.

## Conclusion

In this study, the hybrid coatings containing the graphene nano-sheet (GNS) and nano-hydroxyapatite (nHA) phases have been successfully synthesized on Ti6Al7Nb alloys by a one-step hydrothermal process. The following results can be written from the present study:

- The GNS addition changes the formation and growth mechanism of nHA nucleation.
- SEM observations revealed that the increasing of GNS weight percentage in the layers increases the number of nHA nuclei and surface roughness in the layers and, can be obtained almost an even distribution over the entire surface.
- The synthesized nanocrystalline hydroxyapatite has preferentially flake-like structures. The rod-like structure of nHA was not observed, probably due to the higher content of GNS in the layers in the presented study.
- The EDS analysis showed that the stoichiometric ratio of HA (Ca/P ratio = 1.67) was almost obtained in all GNS ratio.
- The porosities cause a rough on the coated surfaces. However, the nHA formation mechanism is not the only factor affecting surface roughness and the amount of porosity in the layer. The lowest surface roughness was obtained in coatings with 3 wt.% GNS additives. However, it is seen that the surface roughness increases in the samples with 5 and 7 wt.% GNS additives.
- The best hydrophilicity (~52°) property has been obtained in nHA/3GNS coatings. It can be concluded that there is a correlation between the surface roughness and wettability of the coatings.
- The coatings hardness was increased with increasing of the GNS ratio in the coating layers. This behavior can be related to increasing the GNS surface area ratio and the number of nucleated nHAs.
- The in vitro corrosion results showed that the 1 or 3 wt.% GNS addition has a predominant effect on the electrochemical resistance of the coatings.
- The corrosion rates of coatings increased in the following order: nHA/3GNS < nHA/1GNS < nHA/7GNS < nHA/5GNS < only nHA.
